# Structural Positive Electrodes Engineered for Multifunctionality

**DOI:** 10.1002/advs.202404012

**Published:** 2024-07-01

**Authors:** Richa Chaudhary, Amit Chetry, Johanna Xu, Zhenyuan Xia, Leif E. Asp

**Affiliations:** ^1^ Department of Industrial and Materials Science Chalmers University of Technology Hörsalsvägen 7B Göteborg 41258 Sweden; ^2^ Wallenberg Initiative Material Science for Sustainability Department of Industrial and Materials Science Chalmers University of Technology Hörsalsvägen 7B Göteborg 41258 Sweden

**Keywords:** carbon fibre, electrophoretic deposition, lithium‐ion batteries, lithium‐iron phosphate, reduced graphene oxide, structural batteries

## Abstract

Multifunctional structural batteries are of high and emerging interest in a wide variety of high‐strength and lightweight applications. Structural batteries typically use pristine carbon fiber as the negative electrode, functionalized carbon fiber as the positive electrode, and a mechanically robust lithium‐ion transporting electrolyte. However, electrochemical cycling of carbon fibre‐based positive electrodes is still limited to tests in liquid electrolytes, which does not allow for to introduction of multifunctionality in real terms. To overcome these limitations, structural batteries with a structural battery electrolyte (SBE) are developed. This approach offers massless energy storage. The electrodes are manufactured using economically friendly, abundant, cheap, and non‐toxic iron‐based materials like olivine LiFePO_4_. Reduced graphene oxide, renowned for its high surface area and electrical conductivity, is incorporated to enhance the ion transport mechanism. Furthermore, a vacuum‐infused solid‐liquid electrolyte is cured to bolster the mechanical strength of the carbon fibers and provide a medium for lithium‐ion migration. Electrophoretic deposition is selected as a green process to manufacture the structural positive electrodes with homogeneous mass loading. A specific capacity of 112 mAh g^−1^ can be reached at C/20, allowing the smooth transport of Li‐ion in the presence of SBE. The modulus of positive electrodes exceeded 80 GPa. Structural battery‐positive half‐cells are demonstrated across various mass‐loadings, enabling them to be tailored for a diverse array of applications in consumer technology, electric vehicles, and aerospace sectors.

## Graphical abstract

Current challenges in advancing structural batteries involve designing positive electrodes that perform well within a mechanically robust electrolyte. This study overcomes the existing impediments by designing LiFePO_4_‐functionalized carbon fiber‐based positive electrodes and testing them in a biphasic solid‐liquid electrolyte, aka structural battery electrolyte. The multifunctional capability of structural positive electrodes facilitates massless energy storage.

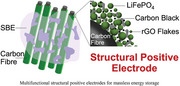

## 1 Introduction

The increasing need for energy, alongside the environmental impacts of CO_2_ emissions mainly from fossil fuel combustion, has driven the search for sustainable energy generation and storage solutions. Particularly, the mobile electronics and automotive sector is shifting toward electric‐powered and lightweight vehicles equipped with batteries to generate and store electric energy. Lithium‐ion batteries (LIB) have played a crucial role in the rapid development of the energy storage industry.^[^ ^1^ ^]^ Despite the development of high‐performance LIBs, their reliance on mono‐functional materials lacking mechanical performance, liquid electrolyte, and rigid protective casing with inadequate load‐bearing capability, renders the battery reliant on inert structural components. This leads to both bulkiness and capacity constraints, limiting their practical use in electric vehicles, electric aircraft, and portable electronics.

Attempts to overcome these impediments have led to the concept of carbon‐fiber‐based structural battery composites that serve both for energy storage and structural integrity and have the potential to replace inert structural components.^[^ ^2^ ^]^ The implementation of structural batteries in electric vehicles and aircraft offers added power along with substantial weight reductions, which can lead to a significant improvement in the energy efficiency of the vehicle.^[^ ^3^ ^]^

In this context, substantial work has been carried out toward the development of current collectors,^[^ ^4^ ^]^ negative electrode,^[^ ^2^, ^5^ ^]^ and various load‐carrying electrolyte systems including solid polymer electrolyte (SPE),^[^ ^6^ ^]^ gel polymer electrolyte (GPE),^[^ ^6^, ^7^ ^]^ two‐phase electrolyte,^[^ ^8^ ^]^ and heterogeneous electrolytes/polymerization induced phase separation (PIPS) electrolyte^[^ ^9^ ^]^ while the positive electrode^[^ ^10^ ^]^ has received comparatively less scrutiny in the regard of multifunctional structural battery. Hence, the current challenge in advancing structural batteries lies in their positive electrode components.

Previous studies have investigated the deposition of lithium iron phosphate (LiFePO_4_, LFP) onto carbon fibers (CF) using multiple dipping methods, resulting in a layer‐by‐layer (LbL) assembly of LFP particles on the CF surface.^[^ ^11^ ^]^ These LFP particles were modified with anionic polyethylene‐imine (PEI) and a cationic dispersion binder of cellulose nanofibrils (CNF). Electrostatic interactions during each dipping cycle facilitated the formation of a bilayer, with subsequent repetitions leading to a multilayer coating. Upon heating to 450 °C, the PEI and CNF underwent carbonization, forming a conductive carbon skeleton containing the active LFP particles. The LbL‐coated CF electrode displayed a specific capacity of ≈100 mAh g^−1^ at 0.1C in liquid electrolyte.^[^ ^11^ ^]^ Fabrication of LFP coating onto CF was also studied using the spray coating technique.^[^ ^12^ ^]^ The slurry composition of 88 wt.% LFP, 6 wt.% carbon black, and 6 wt.% polyvinylidene fluoride (PVDF) binder was explored in N‐Methyl‐2‐pyrrolidone (NMP) solvent while the spraying process was done in batch steps to enhance the absorption of the particles onto CFs, reporting the specific capacity of 123 mAh g^−1^ at the C‐rate of 1.5C in liquid electrolyte.^[^ ^12^ ^]^ Electrophoretic deposition (EPD) has also been used to deposit various proportions of LFP, carbon black (CB), and PVDF onto T800 CF.^20^, ^[^ ^10b^ ^]^ Among these, the sample coated with a ratio of 90:6:4 (LFP: CB: PVDF) exhibited superior performance, delivering a specific capacity of 110 mAh g^−1^ with a capacity retention of ≈50% after 1000 cycles.^[^ ^10b^ ^]^ The performance of EPD‐deposited LFP onto CFs has been further enhanced with an additional coating of nanosheets of electrochemically exfoliated graphene oxide (EGO).^[^ ^10a^ ^]^ This approach allowed the success of uniform coatings with tuneable conductivity. The CFs were deposited with the compositions of 90.6 wt.% LiFePO_4_, 4.5 wt.% EGO, and 4.9 wt.% CB. The performance of deposited composites was tested in a liquid electrolyte, resulting in a specific capacity of 72 mAh g^−1^ at 2C‐rate. The potential of a full cell setup was also demonstrated, using a T800 CF negative electrode and LFP/EGO coated CF positive electrode, separated by a microfiber separator soaked in liquid electrolyte. The cell exhibited a maximum specific capacity of 79.85 mAh g^−1^ at 0.1C, with 30% capacity retention after a 20‐fold increase in rate to 2C.^[^ ^10a^ ^]^

However, the electrochemical performance of the mentioned positive electrodes has only been validated in liquid electrolytes, posing the most prominent challenge to realizing an all‐fiber structural battery.

This work aims to develop an environmentally friendly process for synthesizing CF‐based positive electrodes with graphene additives, to achieve an all‐fibre structural battery composite. Green chemistry principles are being leveraged to advance an approach demonstrated for synthesizing structural positive electrodes.^[^ ^10a^ ^]^ The integration of an electrically conductive graphene/carbon black (CB) scaffold to coat lithium iron phosphate on CF is expected to facilitate rapid ion transport and enhance cycling stability, owing to the resulting high intrinsic electrical conductivity and mechanical flexibility of the electrode composite material. The novelty of the present work includes i) the development of homogeneously coated carbon fibers positive electrodes, ii) proof of concept to test their electrochemical and mechanical performance in structural battery electrolyte (SBE), and iii) the current approach of electrodeposition is based on green solvent ethanol and environmentally friendly LiFePO_4_ particles, reduced toxicity, and cost.

## 2 Results and Discussion

**Figure 1:**
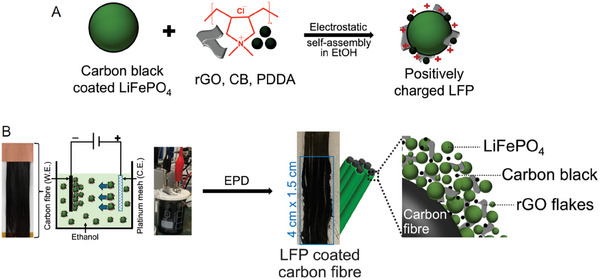
Schematic illustration of electrophoretic deposition (EPD) depicting the integration of LiFePO_4_ onto carbon fibers. A) Electrostatic self‐assembly of positively charged LFP. B) Electrophoretic deposition process and the real‐time sample pictures.

One pot electrophoretic deposition is designed to achieve the high‐loading cathode active materials, LFP on electrochemically inactive carbon fibers for positive electrodes (**Figure** **1**). Graphene has garnered significant interest due to its remarkable attributes, including a high theoretical surface area (2630 m^2^ g^−1^), electrical conductivity (108 S m^−1^), and mechanical stiffness and strength (Young's modulus of 1000 GPa).^[^ ^13^ ^]^ These characteristics position it as an ideal candidate for the scaffolding of LFP on CFs. Highly conductive carbon black is further introduced to enhance the electronic conductive and PDDA is added in catalytic amount to stabilize the EPD solution. Tunable mass loadings of LFP onto CFs are achieved by applying 70 V in direct current mode. The as‐synthesized structural positive electrodes are used to fabricate the pouch cells in half‐cell configuration and tested for their electrochemical and mechanical properties.

### 2.1 XRD Confirms the Crystalline Orthorhombic Phase of LiFePO_4_

**Figure 2:**
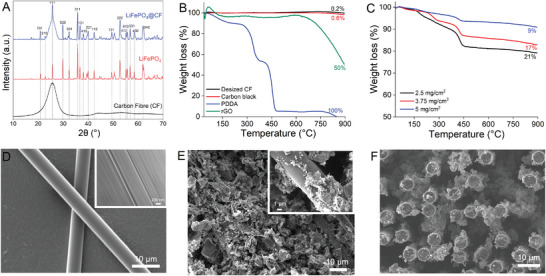
Structural and microscopic characterizations of LFP‐coated positive electrodes. A) XRD represents the crystalline orthorhombic phase of LiFePO_4_. B,C) Thermogravimetric analysis of EPD components and LFP‐coated carbon fibers. D) Scanning electron microscopic (SEM) image of desired carbon fibers. E,F) Surface and cross‐section of LFP deposited carbon fibers.

The structural confirmation of LiFePO_4_ olivine particles deposited on carbon fibers (LFP@CF) is analyzed by the X‐ray diffraction patterns (**Figure** **2**A), confirming the presence of crystalline orthorhombic LiFePO_4_ phase. The experimental peaks are matched with ICDD files and hkl planes are computed with Topas software. The intense crystalline peaks observed at 2𝜃 (hkl) value of 20.0 (101), 22.8 (207), 25.6 (111), 29.9 (020), 32.1 (301), 36.7 (121), 38 (410), 40 (221), 42.4 (112), 49.4 (131), 52.6 (222), 55.1 (610), 56.7 (331), 58.4 (430), and 61.9° (040) match the experimental data for the orthorhombic space group, *Pnma* (Joint Committee on Powder Diffraction Standards, JCPDS no. 40–1499 and  ICDD database, PDF card number 00‐040‐1499), indicating the phase purity in the deposited samples. No additional impurity phases were detected, and there is no appearance of the CB and rGO peaks, suggesting that the EPD process did not generate any impurities. Additionally, the inclusion of a low content of graphene and carbon black did not affect the primary crystal phase of LFP. The broad diffraction peak at 2θ of 25.6° is observed from the amorphous phase of the carbon fiber backbone.

### 2.2 LFP Coated Carbon Fibres Validate the Presence of over 80% Active Material

The amount of LFP coated on the carbon fibers is determined by thermogravimetric analysis. Figure 2B illustrates the TGA pattern of desired CF, CB, rGO, PDDA, and Figure 2C represents varying loadings of LFP‐coated CFs. A slight weight loss temperature below 150 °C in the pure and coated samples could be associated with water content. The weight loss at 150 °C is higher in PDDA (8%), which is due to the presence of water content in commercial samples. PDDA exhibits complete weight loss at 450 °C while the graphitic sheets in rGO start degrading above 600 °C. The LFP functionalized CFs exhibit one main weight loss stage between 150 and 450 °C, which is associated with the removal of physically absorbed PDDA molecules from the surface of CFs. The total weight loss for 2.5, 3.75, and 5 mg cm^−2^ samples is found to be 21, 17, and 9% (starting weight % at room temperature – end weight % at 800 °C), respectively. The active material present on the carbon fibers is calculated based on TGA results and used to calculate the specific capacity of each sample.

### 2.3 SEM Analysis Substantiates the Uniform Deposition of LFP onto CFs

The surface morphology and coating quality of desired and LFP‐functionalized carbon fibers were examined using scanning electron microscopy (SEM). The SEM image of desired CFs (Figure 2D) confirms the thorough washing of CFs, with no visible traces of polymer sizing remaining on the CFs. Additionally, the presence of grooves on carbon fibers is clearly visible under a magnification of 200 nm (Figure 2D inset). The morphology observed for all the electrophoretically deposited samples appears similar. Figure 2E, depicts the surface and cross‐section SEM images of the LFP‐coated CFs. Following EPD, the LFP/EGO/CB coating covers the entire surface of each CF, with well‐dispersed LFP particles observed within the coating. These particles are either enveloped or partially covered by EGO flakes, and there are no visible detached fragments or cracks observed under SEM analysis.

### 2.4 An Increase in LFP Loading leads to a Decrease in Electrochemical Performance and Charge Storage in Structural Half‐cell Composite Electrodes

**Figure 3:**
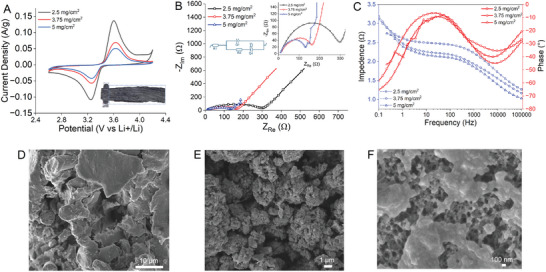
Structural battery electrolyte (SBE) infused positive electrodes. A) Cyclic voltammetry represents the characteristic peak of LiFePO_4_ redox reaction. B,C) Electrochemical Impedance Spectroscopy of LFP coated carbon fibers. D–F) SEM of SBE infused positive electrode at varying magnifications, representing the porous structure.

The impact of various LFP loadings on the electrochemical behavior of structural half‐cells was investigated using cyclic voltammetry (CV). In **Figure** **3**A, the CV of samples coated with 2.5, 3.75, and 5 mg cm^−2^ loadings is depicted, with a scan rate of 0.1 mV s^−1^ in a voltage range of 2.6 V–4.2 V versus Li+/Li. All the electrodes, fabricated by the EPD method, represent well‐defined redox peaks in the range of 3.20 V‐3.60 V versus Li/Li^+.^ This typical oxidation/reduction peaks during the anodic/cathodic scan attributed to the reversible faradaic redox reaction related to the Li‐ion intercalation and deintercalation processes (Fe^2+^/Fe^3+^) in LiFePO_4_ crystal structure, consistent with prior findings.^[^ ^10a^ ^]^ However, notable differences were observed in peak shapes, current densities, and potential intervals between the 2 redox peaks. The 2.5 mg cm^−2^ electrode composite sample displayed sharp and symmetric peaks with a small potential difference (≈0.33 V) between the anodic and cathodic peaks, indicative of low polarization and high redox reversibility. Conversely, the higher loadings of 3.75 and 5 mg cm^−2^ exhibited slightly broader and less intense peaks with a similar potential difference (0.33 V). This observation aligns with TGA results, which confirmed a higher weight percentage of poorly conductive LFP in the 5 mg cm^−2^ samples, leading to the least intense peak. The area under the CV curves directly correlates with the stored charge amount, with the lowest loading sample showing the highest specific currents and area under the curve compared to samples with higher loadings.

### 2.5 The Lowest Interface Resistance of 7 Ω between the Electrolyte and Electrode Surface in SBE is Achieved by Vacuum Infusion Technique

Electrochemical impedance (EIS) measurements were conducted to delve deeper into the electrochemical performance and electrode processes of LFP‐deposited CFs SBE composites. Figure 3B represents the Nyquist plot of deposited samples. The Spectra of all 3 samples exhibit a semicircle in the high‐frequency region and a linear trend in the low‐frequency region, fitting well with an equivalent circuit shown inset of Figure 3B. The components of this circuit include electrolyte and electrode resistance (R1), charge transfer resistance (R2), and constant phase element (Q).  The semicircles have a high‐frequency intercept representing the ionic conductivity of electrolyte (*R*_s_) while the semicircle's diameter corresponds to the charge transfer resistance (R2) at the electrode‐electrolyte interface. The linear segment at low frequency is attributed to lithium‐ion diffusion within the electrode, reflected by the Warburg resistance. The impedance is also illustrated as a Bode diagram (Figure 3C) with separate magnitude and phase responses. The advantage of using the Bode diagram lies in its inclusion of frequency data, a feature absent in the Nyquist plot. However, despite this distinction, the Bode diagram still offers insights into impedance behavior, with phase behavior outlining the boundaries for each region. In contrast to the 5 and 3.75 mg cm^−2^ samples, the reduced *R_ct_* in the 2.5 mg cm^−2^ sample indicates a faster electrolyte‐electrode interaction. This reduction in resistance is attributed to enhanced electronic and ionic conductivity in the 2.5 mg cm^−2^ electrode, leading to significant improvements in electrochemical performance, as the faradaic process primarily relies on electron conduction and ion transfer. Moreover, SEM micrographs validate the porous structure of SBE‐infused positive electrodes. Prior to SEM imaging, all samples undergo a 24‐h water soaking followed by 12‐h vacuum oven drying to eliminate any remaining liquid. Subsequently, the dried positive electrodes reveal a porous morphology with a diverse spectrum of pore sizes, including those below 100 nm (Figure 3E,F).

### 2.6 The SBE‐Infused Positive Electrodes Demonstrate a High Transference Number of 0.6

**Figure 4:**
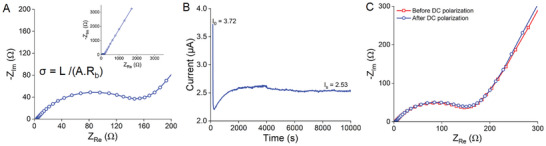
Ionic Conductivity and lithium‐ion transference number in SBE‐infused positive electrodes. A) Nyquist impedance plot at room temperature B) Current‐time curve obtained from chronoamperometry. C) Impedance plot before and after DC polarization.

The lithium transference number is the number of moles of lithium ions transferred per one Faraday of charge transferred. In a high‐conductivity lithium polymer battery, ideally, the lithium transference number should approach unity, which can be simply calculated by dividing the initial current (*I_s_*) by the steady‐state cell current (*I_ss_*). However, the current system is more complex due to small traces of impurities, water, or solvent molecules on the electrodes, which can potentially alter experimental results. Hence, a correction factor is introduced to assess the system's characteristics before and after polarization.^[^ ^14^ ^]^ A Bruce‐Vicent method is used to determine the lithium transference numbers in structural battery electrodes. Since, the present work is focused on structural positive electrodes, where the electrolyte is integrated within the electrode, there is no need for additional liquid electrolyte or an electrolyte film in the battery. Therefore, SBE‐infused structural positive electrodes are directly utilized for all calculations. **Figure** **4**A depicts the Nyquist plot of the structural positive electrode. The bulk resistance, *R_b_*, is obtained from the high‐frequency intercept of the impedance spectra and ionic conductivity is calculated to be 7.4 × 10^−4^ S cm^−1^, which is in the same order of magnitude reported for neat SBE.^[^ ^15^ ^]^

The initial and steady‐state current is acquired from chronoamperometry measurement (Figure 4B), while the bulk resistance of the structural positive electrode before (172 Ω) and after (181 Ω) DC polarization is calculated by fitting the Randles circuit R1+Q1/(R2+Q4) (Figure 4C). The structural positive electrode reveals a high lithium transference number (t_Li+_) of 0.55, indicating a substantial contribution of Li+ ions to the total ionic conductivity.

### 2.7 Highest Specific Capacity of 122 mAh g^−1^ and 99.6% Capacity Retention is Achieved

**Figure 5:**
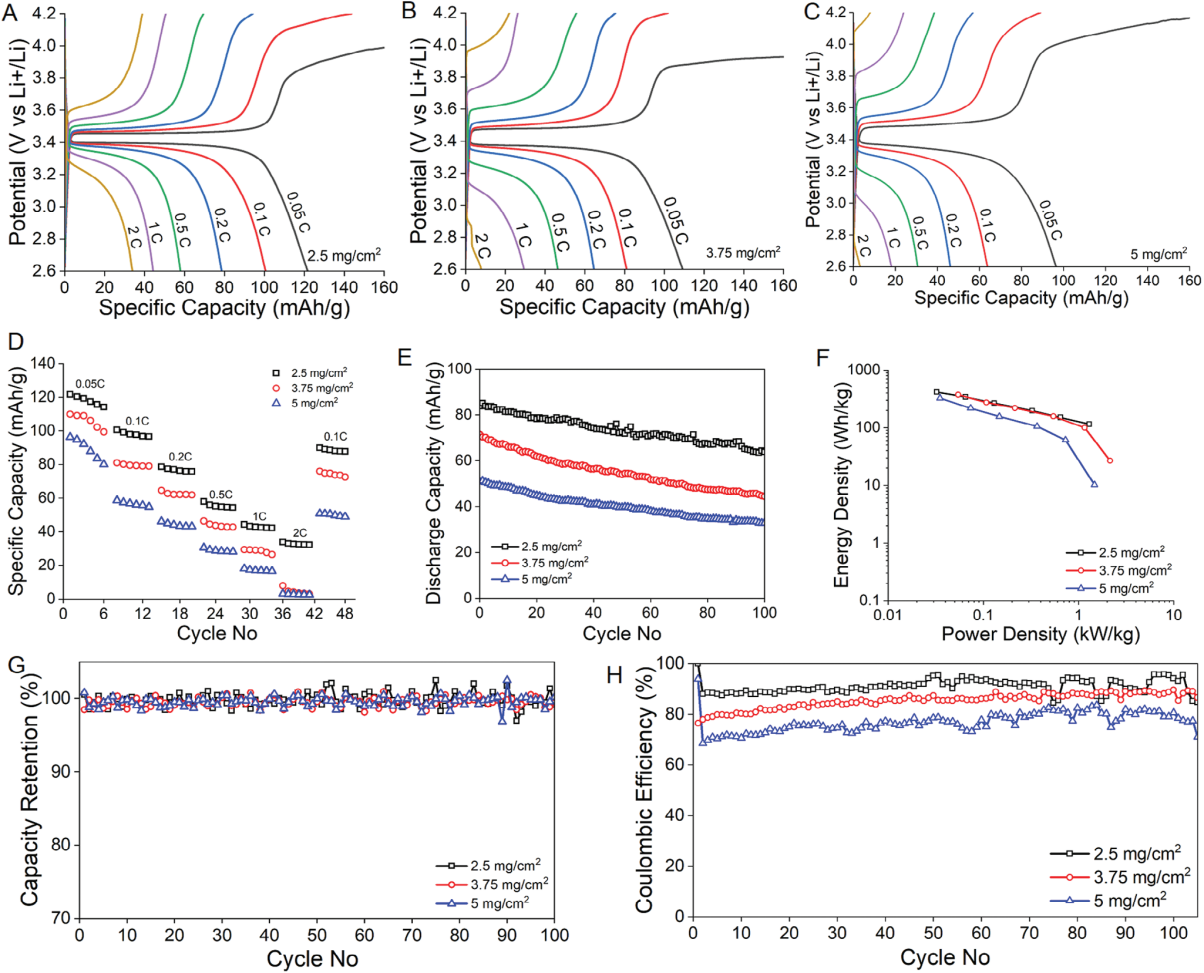
Galvanometric cycling of positive electrodes of varying mass loadings in SBE. A) 2.5 mg cm^−2^. B) 3.75 mg cm^−2^. C) 5 mg cm^−2^. D) Specific capacities at varying C‐rates for 6 cycles each. E) Discharge capacity of all loadings for 100 cycles. F) The Ragone plot represents the energy and power density. G) High‐capacity retention confirms the long‐term cycling stability. H) Stable columbic efficiency of positive electrodes.

The SBE‐infused positive electrodes are cycled in a half‐cell lamina and subjected to charge‐discharge cycling at 0.05, 0.1, 0,2, 0.5, 1, and 2C rates. **Figure** **5** illustrates the specific capacities of the cells at a potential span of 2.6–4.2 V (Figure 5A–C) for different loadings of LFP. The electrochemical performance of the cell with 2.5 mg cm^−2^ reveals the highest specific capacity of 122 mAh g^−2^ at 0.05C and the longest discharge time. The average plateau was ≈3.39 V versus Li^+^/Li for all samples, corresponding to the Fe^2+^/Fe^3+^ redox reaction. The polarization between the charging and discharging plateaus for lower to higher loading samples is calculated to be 0.06 V, 0.11 V, and 0.15 V 2.5, 3.75, and 5 mg cm^−2^, respectively. The lowest polarization of 0.06 V for 2.5 mg cm^−2^ was attributed to low internal resistance, in agreement with the cyclic voltammogram (Figure 3A). A decrease in the specific capacity by increasing the C‐rate from 0.05C to 2C is observed for all the samples. Specific capacities of structural positive electrodes at different C‐rates as a function of the cycle number are presented in Figure 5D. The sample with lower loading of active material 2.5 mg cm^−2^ exhibited the best rate capability among all, delivering a discharge capacity of 101 mAh g^−1^ at 0.1C, which further decreased to 81 and 64 mAh g^−1^ for 3.75 and 5 mg cm^−2^ samples, respectively. This phenomenon is supported by EIS measurements indicating lower overall resistance (Figure 3B). Furthermore, it is presumable that higher loading leads to an increase in the thickness of deposition, which elongates the diffusion path required for the electrolyte to access the active material. In other words, the resistance increases for the electrolyte diffusion to internal pores along with the thickness increase. Moreover, it is evident from charge‐discharge profiles that the charge capacity for higher‐loading samples exceeds the discharge capacity, accompanied by substantial polarization and limited reversibility. This phenomenon results from the higher consumption of lithium during the formation of solid‐electrolyte interphase (SEI) at the beginning of the charging process. However, in subsequent cycles, the reversible capacity emerges which confirms the stable capacity delivered by the electrode after the formation cycles are completed. Although the capacity of all loadings decreases with increasing C‐rate; however, this process is reversible, and the initial capacity was restored by returning to 0.1C after rate cycling. The capacity of the higher‐loading samples is observed to be the lowest for all the C‐rates, suggesting that not all the deposited LiFePO_4_ is accessible for the ions. The monitoring of discharge capacity across multiple cycles provides insights into battery degradation, efficiency, and overall health. The discharge capacity of all samples is tracked over 100 cycles and represented in Figure 5E.

The energy density and power density could be calculated from the charge‐discharge profile and are presented in the Ragone plot (Figure 5F). The latter is usually used to evaluate the capacitive performance by correlating energy density as a function of power density. The sample with a lower loading of LFP demonstrated a remarkable specific energy density of 342 Wh kg^−1^ paired with a specific power of 1.6 kW kg^−1^. However, as the loading increases from 3.75 to 5 mg cm^−2^, the energy density gradually diminishes to 275 and 218 Wh kg^−2^ for a 0.1C rate, corresponding to power densities of 2.6 and 1.8 kW kg^−1^, respectively. This decline aligns with the trend observed in the CV and GCD curves, indicating that the increase in loading thickness restricts access to internal pores, thus reducing energy density. The performance matrix of structural positive electrodes needs to be compared with the commercial LFP batteries. However, all the traditional LFP batteries are based on a liquid electrolyte system which has 10‐fold higher ionic conductivity (1 M LiTFSI: 4.3 × 10^−3^ S cm^−1^) than the SBE (SBE, 2.9 × 10^−4^ S cm^−1^).^[^ ^15^ ^]^ This lower ionic conductivity of SBE significantly limits the capacity of the structural battery and hence it results in an unfair comparison of present work with liquid electrolyte‐based LFP batteries.

Long‐term cycling stability is tested for all the samples at a 0.1C rate for 100 cycles. Capacity retention of ≈99.6% is achieved for all the samples (Figure 5H) —indicating efficient energy conversions and delivery of capacity. However, the Columbia efficiency is observed to be in the range of 90%, 80%, and 70% for samples 2.5, 3.75, and 5 mg cm^−2^, respectively (Figure 5G). This decline can be attributed to the half‐cell design and the presence of metallic lithium as a negative electrode. As the number of cycles increases, the inactive layer on the lithium grows thicker, adding extra resistance in the cells and slowing down fast kinetics.

Essentially, the stable battery performance of the 2.5 mg cm^−^^2^ sample may stem from the improved conductivity attributed to the thin coating on carbon fibers, allowing for better accessibility of ions to most LFP particles. The presence of carbon black and graphene oxide further enhances this effect. However, as the loading increases, higher resistance is observed from CV measurements, attributed to the reduced surface area and active site density. Additionally, the increased charge transfer resistance with higher loading may result from changes in the lithiation degree of the electrodes, leading to a loss of cyclable lithium. Another factor is the weak penetration of the electrolyte into the densely loaded active material, reducing lithium ion diffusion at the interface due to the low ionic mobility of the electrolyte. This hinders lithium ion migration to the electrode, significantly increasing charge transfer resistance at higher loadings.

This is further supported by the charge‐discharge profiles in Figure 5A–C, as the loading of active material increases, the capacity decreases, and the electrical polarization increases. This behavior is due to reduced lithium ion diffusivity within the LFP electrode and increased charge‐transfer resistance at the electrode‐electrolyte interface, leading to lower electrochemical reactivity and poor kinetics. This indicates a blockage of lithium‐ion transmission between the electrolyte and the interface. Consequently, the electrolyte poorly penetrates the active materials, slowing the electrochemical reaction rate. The sluggish kinetics cause an excess of active lithium ions that cannot participate in the reaction or diffuse through the SBE electrolyte, leading to irreversible reactions and side reactions that generate impurities. These impurities accumulate on the cathodes, forming a passivation layer that grows during charging, resulting in slow lithium ion diffusion, reduced conductivity, and significant battery capacity loss.

Furthermore, the Coulombic efficiency decreases with increased active material loading, indicating poor reversibility. This decrease, along with cell aging, is due to a loss of cyclable lithium. Additionally, the low diffusivity of lithium ions at higher loadings affects the stability of the interface between the cathode and electrolyte, accelerating capacity fade.

### 2.8 Analysis of Cycled Electrode Demonstrates an Increase in Charge‐Transfer Resistance

**Figure 6:**
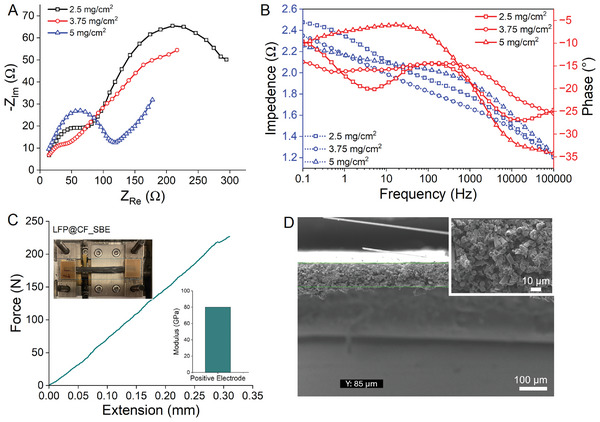
Post‐cycle analysis of structural positive electrodes. A,B) Nyquist and Bode plot for cycled electrodes. C) Modulus of SBE‐infused cycled electrode in tensile mode D) SEM cross‐section of SBE‐infused positive electrode.

Since all the electrodes are loaded with different amounts of LFP, it is essential to test their performance after charge‐discharge cycling. The post‐cycling EIS spectra is performed on fully charged cells and presented in **Figure** **6**A,B. The intercept on the real axis in the high‐frequency region (Rs) of the EIS spectra is comparable for all samples (14 Ω), mainly originating from electrolyte and electrode resistance, indicating smooth Li‐ion migration. However, a significant increase in the electrode‐electrolyte interface resistance is observed for the lower LFP loading sample (2.5 mg cm^−2^).

The semicircle at high‐to‐medium frequencies indicates the charge transfer resistance (*Rct*) of the reaction and it is significantly increased for 2.5 mg cm^−2^ sample compared to 3.75 (28 Ω) and 5 mg cm^−2^ (56 Ω) samples, suggesting a hindered interfacial redox reaction process during the charge‐discharge process in the lowest loading sample. This increased *Rct* agrees with TGA results, which show the highest amount of PDDA is present in the lowest loading sample – possibly creating an insulating layer in the electrode. Notably, a rapid electrolyte‐electrode complex reaction is observed for all the cycles. Since the faradaic process is mainly determined by electron conduction and ion transfer, the low resistance might be ascribed to the appropriate electronic and ionic conductivity of the LFP‐deposited CF electrodes.

### 2.9 The Positive Electrode Achieves a High Modulus of 80 GPa

The mechanical properties of the structural battery composites before cycling were characterized under tensile loading (Figure 6C). Precise assessment of composite strength demands meticulous sample preparation, including the polishing of free edges and tabbing clamping regions to prevent premature failure and, consequently, the underestimation of material strength. However, undertaking such sample preparations was not achievable here, furthermore, the dimensions of the positive electrode do not allow measuring strain without the option to employ conventional techniques like digital image correlation or strain gauges. Additionally, the micro tensile stage employed for micro testing posed a challenge in adhering to conventional test standards (ASTM D3039). Hence, to obtain reliable measurements system/machine compliance was calculated and subtracted from apparent compliance to yield the actual specimen compliance. The thickness of SBE infused positive electrode is measurement by SEM (Figure 6D) and a typical force‐extension curve is presented in Figure 6C. A minimum of 5 samples are tested and average modulus of 80 GPa is achieved.

## 3 Multifunctional Performance and Efficiency Offer Potential Weight Savings

Carbon fiber‐based positive electrodes to date have been demonstrated and characterized in liquid electrolytes.^[^ ^10^, ^16^ ^]^ In the current study, the liquid electrolyte is replaced by a structural electrolyte matrix material providing a general multifunctional performance to the electrode. That is, the SBE matrix provides mechanical load transfer, also in shear and compression, as well as ion conductivity between fibers in the electrode. The multifunctional performance of the electrode material is assessed by its multifunctional efficiency metrics. In this approach, the electrochemical efficiency (η_e_) is defined as the ratio of the specific capacity of the structural positive electrode to that of a conventional LFP cathode.^[^ ^17^ ^]^ Similarly, the structural efficiency, η_s_, is the ratio of the tensile modulus of the structural positive electrode and the monofunctional carbon fiber composite. The multifunctional efficiency is η_mf_ = η_e_+η_s_. A mass reduction can be realized if the multifunctional efficiency exceeds unity.

The electrochemical efficiency, ηe, of the structural positive electrode, is 0.66, given its specific capacity of 112 mAh g^−1^ relative to the standard theoretical capacity of LFP (170 mAh g^−1^).^[^ ^18^ ^]^ Furthermore, its structural efficiency, η_s_, is 0.49, relating its measured tensile modulus of 80 GPa to that of a monofunctional T800 reinforced polymer composite with a fiber volume fraction of 55% (with a tensile modulus of 163 GPa). Consequently, the structural positive electrode offers mass savings as its multifunctional efficiency, η_mf_, is 1.15.

## 4 Conclusion

The advancement of carbon fiber‐based structural positive electrodes employing SBE represents a significant leap in energy storage technology. By integrating the dual functionalities of load bearing and ion transport within the electrolyte, these batteries offer a pathway to energy storage without adding mass, opening new avenues for lightweight, high‐strength applications. Utilizing cost‐effective iron‐based materials for electrodes, coupled with graphene to enhance ion transport, ensures both affordability and performance. Additionally, the incorporation of vacuum‐infused solid‐liquid electrolytes enhances mechanical properties, enabling the structural batteries to endure demanding conditions. With specific capacities achieving 112 mAh g^−^ and impressive Young's modulus of 80 GPa, these structural positive half‐cells showcase promising potential across a variety of industries. As research in this field progresses, structural batteries stand poised to transform energy storage solutions, providing tailored options to meet diverse application requirements.

## 5 Experimental Section

### Materials

Polyacrylonitrile (PAN)–based carbon fibers of the type T800SC‐12K‐50C were manufactured by Toray Composite Materials America, Inc. These fibers were then processed into ultrathin unidirectional (UD) tapes, ≈15 mm wide, by Oxeon AB, Sweden. Lithium iron phosphate (LiFePO_4_) powder, with an average particle size of 3.5 µm, was obtained from MTI Corporation. Reduced graphene oxide (rGO) with a carbon content of 95% was sourced from LayerOne AS, Norway. Additionally, conductive super P carbon black (CB, ≈40 nm) and polydiallyldimethylammonium chloride (PDDA; 20 wt.% in H_2_O) were purchased from Thermo Scientific and Sigma Aldrich, respectively. Absolute ethanol (≥99.8%) was acquired from Avantor VWR Sweden, and the Whatman glass microfiber separator (Whatman GF/A, 260 µm thick) was supplied by Sigma Aldrich. The constituents of the bi‐continuous SBE included bisphenol A ethoxylate dimethacrylate (BPAMA) (Mn: 540 g mol^−1^) monomer supplied by Sartomer Company, Europe, and the heat‐initiator 2,2′‐azobis(2‐methylpropionitrile) (AIBN), lithium bis(trifluoromethanesulfonyl)imide (LiTFSI, anhydrous 99.99%), propylene carbonate (PC) (PC ≥ 99%, acid <10 ppm, H_2_O <10 ppm), and ethylene carbonate (EC) (99% anhydrous), all obtained from Sigma Aldrich. Additionally, acrylic binder base PELCO conductive carbon glue, used for bonding the current collector and carbon fibers, was purchased from Caspilor, Sweden.

### Methods: Desizing of Carbon Fibres

Commercially obtained carbon fiber is often coated with a layer of polymer sizing, which is applied during the manufacturing process. This sizing not only shields the fibers but also improves their mechanical and handling characteristics. However, this polymeric coating creates an insulating layer on fibers which needs to be removed for better electrochemical properties and homogeneous deposition. The desizing of carbon fibers was achieved by refluxing the carbon fibers in dichloromethane for 8 h.

### Synthesis of Structural Positive Electrode by Electrophoretic Deposition

The high‐voltage EPD process was conducted in a two‐electrode compartment under ambient conditions, with a constant voltage applied by a Keithley 2450 Sourcemeter power supply. Desized carbon fiber served as the working electrode (WE), with an area of 60 x 15 mm submerged in the solvent, while a platinum mesh (80 mm long and 20 mm wide) functioned as the counter electrode in parallel orientation. The distance between the working and counter electrodes was fixed at 30 mm. A deposition solution consisting of 5 mg mL^−1^ LFP dispersion in ethanol, with the addition of rGO and CB, was utilized. The applied voltage was set to 70 V, and the deposition time was fixed at 20 min, based on prior investigations.^[^ ^10^, ^16^ ^]^ A schematic illustration of the EPD process is presented in results, and discussion section (Figure 1).

The desired CFs were spread to a width of 1.5 cm and tabbed at the ends to prevent any short circuits during the EPD process. Preparation of EPD bath starts by dispersing 500 mg of LiFePO_4_ powder in 50 mL of ethanol using a Sonics VCX‐750 Vibra‐Cell ultrasonic liquid processor for 20 min. Subsequently, 50 mg of reduced graphene oxide (rGO) and 50 mg of carbon black dispersed in 50 mL of ethanol were added to the dispersion as carbon‐based conductive additives. Since both rGO sheets and CB carry negative charges in ethanol, the surface charge in the solution was adjusted by gradually adding 500 µL of PDDA dissolved in a small amount (1 mL) of ethanol and subjected to tip sonication for 20 min. PDDA, a cationic polymer commonly used for particle surface charge tuning, was employed here in a catalytic amount (0.1 wt.% of LFP). This addition of PDDA helps to modify the surface charge of CB and rGO to a positive state, allowing for their electrostatic adsorption onto the surface of negatively charged LiFePO_4_ particles. The final suspension was subjected to a cathodic deposition at room temperature which contains 90 wt.% LiFePO_4_, 5 wt.% rGO, and 5 wt.% CB. All samples were synthesized in a similar fashion with 90 wt.% of active material, LFP deposition, and stored in an oven at 70 °C. Prior to pouch cell fabrication, all synthesized samples were characterized for their structural and morphological properties using X‐ray diffraction (XRD), thermogravimetric analysis (TGA), and scanning electron microscope (SEM) techniques.

### Synthesis of SBE

The SBE mixture was prepared following the reported procedure with slight adjustments.[9b] Initially, a stock solution of 1 M liquid electrolyte and SBE was prepared in a glovebox, ensuring an argon atmosphere and dry conditions (<1 ppm H_2_O, <1 ppm O_2_). The liquid electrolyte solution is made from 1.0 M LiTFSI dissolved in EC:PC 1:1 (50:50 wt.%).

For the SBE solution, liquid electrolyte (1 M LiTFSI in EC.PC (50:50)) was mixed with BPAMA monomer and the heat‐initiator AIBN (1 wt.% of the BPAMA monomer). The mixture was stirred vigorously to ensure homogeneity using a vortex and then left inside the glovebox for degassing before being used for vacuum infusion. The vacuum‐infused SBE was cured at 90 °C for 45 min in oven.

### Manufacturing of Structural Battery in Half Cell Configuration

Carbon fiber composite positive electrodes were prepared using a vacuum infusion process, followed by assembly into half‐cells within a pouch bag. Aluminium (Al) foil current collectors were affixed to one end of the LFP‐coated carbon fibers using carbon glue. The vacuum bag assembly was set up on a glass plate, starting with the placement of release plastic to prevent contamination and electrode sticking. Subsequently, the LFP‐coated positive electrodes connected to the Al current collectors were positioned on the release plastic. The setup was covered with perforated polyethylene film and a breather fabric. Silicon tubes (4 mm) were connected for inlet and outlet, sealed with a vacuum bag and rubber tape, and dried in a vacuum oven at 50 °C for 12 h. The SBE was prepared in an argon atmosphere dry glovebox and filled into a syringe. The syringe was connected to one side of the silicon tube inside the glovebox, sealed with clamps, and infused at 0.5 bar pressure regulated by a vacuum valve. After infusion, the system was cured at 90 °C for 45 min. The cured LFP‐deposited carbon fiber lamina was then sealed in a two‐electrode pouch bag made of PET:Al:PE (12:9:75 µm). Lithium metal foil served as the counter and reference electrode with a nickel current collector, and a Whatman glass‐microfiber filter was used as a separator between the electrodes. To facilitate ionic conduction, a small volume (≈200 µL) of supplementary liquid electrolyte was added to wet the separator film.

### Structural, Thermal, and Morphological Properties of Positive Electrodes

X‐ray diffraction (XRD) is carried out with a Bruker D8 Advance powder diffractometer, using Cu‐Kα radiation operated at 45 kV and 40 mA, in the range from 2𝜃 = 10° to 70°, at a step size of 0.2 °/s. Phase identification is performed by matching diffraction peak positions and relative intensities to reference JCPDS files. Thermal degradation of the EPD bath components and LFP deposited CFs are measured using thermogravimetric analysis (TGA). TGA is carried out using a TGA/DSC3+ Mettler Toledo instruments over a temperature range from 30 to 900 °C at a ramp rate of 10 °C min^−1^ under nitrogen atmosphere. Scanning electron microscopy (SEM) was employed to examine the quality of the EPD‐deposited carbon CFs. SEM was performed using JEOL JSM‐7800F Prime model at an acceleration voltage of 5 kV and gold coating of 5 nm.

### Ionic Conductivity and Li‐Ion Transference in Structural Positive Electrode

The SBE‐infused positive electrode was sandwiched between 2 lithium foils and sealed in a pouch bag under an argon atmosphere. Impedance measurements were conducted between 1000 kHz and 0.01 mHz. The ionic conductivity was then calculated using the formula σ  = * L */(*A*.*R*_*s*_), where L represents the thickness of the SBE‐infused positive electrode (80 µm), A is the area of the electrodes (3 cm^2^), and R_s_ is the bulk resistance of the SBE infused positive electrode, which was determined from the high‐frequency intercept of the Nyquist plot.

The lithium‐ion transference number was calculated using Bruce Vincent method by conducting AC impedance tests in conjunction with a DC polarization step. A voltage amplitude of 10 mV was applied until a steady‐state current was achieved (≈3 h). Electrochemical impedance spectroscopy (EIS) was performed and recorded over a frequency range from 1000 kHz to 0.01 mHz before and after DC polarization. The lithium‐ion transference (t_Li+_) number was calculated using the following equation:

(1) $$t_{Li+}\;=\;I_{ss}\left(\Delta V-I_{0}R_{0}\right)/I_{0}\left(\Delta V-I_{ss}R_{ss}\right)$$

where, ΔV is the potential difference applied in the chronoamperometric step. *I_0_* and *I_ss_* are the initial and the steady‐state currents, respectively, and *R_0_* and *R_ss_* are the initial and the steady‐state interfacial resistances. Equivalent circuit modeling was used to determine the interfacial resistances from EIS data.

### Electrochemical Testing of Half Cells

Cyclic voltammetry (CV), galvanostatic charge/discharge (GCD), and electrochemical impedance spectroscopy (EIS) were performed using Bio‐Logic SP‐300 station. CV was conducted at the scan rate of 0.1 mV s^−1^, ranging from 2.6 to 4.2 V versus Li/Li^+^. GCD cycles were performed over the same voltage range, at various rates, starting from 0.05C to 2C for 6 cycles each. The cycling stability was monitored for up to 100 cycles at 0.1C. The selected current for the GCD cycling was calculated from the theoretical capacity of the LFP. EIS measurements were carried out across a frequency range from 100 kHz to 100 mHz using an alternating current (AC). The specific capacity of the samples was determined from discharge curves as per the formula Q=∫Idt/m, where *Q* is the specific capacity in mAh/g based on the deposited active materials mass, *I* is the current, and *m* is the mass of the electrodeposited material (g) and *dt* is the time differential.

### Mechanical Testing of SBE Infused Positive Electrode

For mechanical testing, the cured LFP‐deposited carbon fibre lamina was cut into pieces with dimensions ≈40 mm long and 3 mm wide. Glass fibre tabs was connected to the longitudinal ends of lamina with the help of epoxy adhesive films. This ensures the tight gripping of sample at clamping regions and even load introduction, which prevents the premature failure or gliding of sample. Tensile tests were made on a minimum of 5 samples and the thickness of samples was measured by SEM. The mechanical testing of SBE cured positive electrodes (≈85 µm thick) was performed in tensile mode parallel to the fibre direction using Deben microtester with a 2.0 kN load cell and a testing speed of 0.2 mm min^−1^. Applied strains are extracted from the crosshead displacement of the micro tester and compliance compensation method (ASTM D3379) is used to measure the modulus.^[^ ^2^ ^]^ Briefly, an apparent modulus, *E* is calculated from *E * =  *L*/*CA*, where *L* corresponds to specimen gauge length, *A* is the cross‐section area of the specimen and *C* is the true compliance. The true compliance, C is calculated from *C * = *C*_*a*_  − *C*_*s*_, where *C_a_* refers to the apparent compliance derived from the initial linear segment of the load‐displacement curve, while *C_s_* represents the system compliance, determined experimentally.

### Statistical Analysis

All the presented data were collected from individual samples and independent measurements without any additional processing. Figures were analyzed and plotted using ORIGINPRO 2024 software. The reproducibility of the data was confirmed by performing electrochemical characterizations in triplicate, with one set of results presented in the manuscript.

## Conflict of Interest

The authors declare no conflict of interest.

## Data Availability

The data that support the findings of this study are available from the corresponding author upon reasonable request.
